# Age-Related Mercury Contamination and Relationship with Luteinizing Hormone in a Long-Lived Antarctic Bird

**DOI:** 10.1371/journal.pone.0103642

**Published:** 2014-07-29

**Authors:** Sabrina Tartu, Paco Bustamante, Aurélie Goutte, Yves Cherel, Henri Weimerskirch, Jan Ove Bustnes, Olivier Chastel

**Affiliations:** 1 Centre d’Etudes Biologiques de Chizé (CEBC), UMR 7372 CNRS-Université de la Rochelle, Villiers-en-Bois, France; 2 Littoral Environnement Société (LIENSs), UMR 7266 CNRS-Université La Rochelle, La Rochelle, France; 3 Norwegian Institute for Nature Research, FRAM – High North Research Centre on Climate and the Environment, Tromsø, Norway; University of Lethbridge, Canada

## Abstract

Seabirds, as long-lived top predators, accumulate contaminants such as mercury (Hg), an established endocrine disruptor. In long lived species hormonal secretion varies with age; therefore, Hg-induced endocrine disruption may be exacerbated in some age classes. Here we investigated relationships between blood total Hg and luteinizing hormone (LH, a key pituitary hormone for the onset of breeding), in pre-laying known-age (11–45 years old) snow petrels (*Pagodroma nivea*) from Adélie Land, Antarctica. We predicted that 1) blood Hg would increase with advancing age as a consequence of bio-accumulation; and that 2) increasing blood Hg would be related to decreased concentrations of LH in the most Hg-contaminated individuals. Hg concentrations were higher in females than in males (p<0.001), and contrary to our prediction, decreased with advancing age in males (p = 0.009) and tended to do so in females (p = 0.06). The analysis of stable isotopes (δ^13^C and δ^15^N) suggested that this unexpected pattern could originate from age and sex-related variations in trophic niche, and hence Hg exposure. Regarding LH, our prediction was only supported in young birds (≤23 years) where baseline LH was inversely correlated with Hg concentrations (p = 0.04). Hg burden did not predict baseline LH or GnRH-induced LH in birds that were more than 23 years old. These results show that age and contaminants may interfere with major endocrine mechanisms and, together with other recent studies, support the view that Hg could be connected to LH secretion and could then impair the fitness of long-lived birds.

## Introduction

Mercury (Hg) is a ubiquitous contaminant, present in aquatic biota with elevated concentrations in fish, some mammals and seabirds [1], [2]. Diet is the principal route of contamination: Hg bio-accumulates into individuals [3], [4], [5], [6] and bio-magnifies along the food web [7], [8]. Seabirds are top predators which often bear elevated levels of Hg, and are thus considered good bio-monitors to describe Hg bio-accumulation and bio-magnification in the marine environment [9]. Seabirds are also long-lived organisms, and therefore would accumulate Hg in their tissues over a long life-span [10]. Although it has been well established that seabird nestlings show lower Hg concentrations than adults [11], very few studies have examined the influence of age on Hg contamination in adults and the patterns are not clear [12], [13], [14], [15], [16]. In this study, we report the results of a cross-sectional study on known-age adult snow petrels *Pagodroma nivea*, a small (≈400 g) but very long-lived Antarctic seabird (≈50 years) [17]. We made use of an exceptional long-term banding study (1964- to present, [17]) to identify the age of each bird and measured blood total Hg concentrations. Blood Hg concentrations reflect dietary exposure over the last several weeks and also a complex equilibrium between ingestion, deposition and mobilization [10], [18], [19]. In seabirds, blood Hg is strongly correlated to Hg concentrations in storage organs [20], [21], [22]; blood Hg is thus considered as a good proxy of overall Hg burden. Given the ability of Hg to bio-accumulate we would expect blood Hg concentrations to be positively related to age in adult snow petrels. As mentioned previously, diet is the principal route of Hg contamination and to further interpret the possible Hg/age relationship, we used the isotopic niche as a proxy of the trophic niche, with δ^13^C and δ^15^N values indicating the birds’ foraging habitats and trophic position, respectively [23]. Indeed this possible Hg/age relationship could originate from an age-related change in trophic niche. Snow petrels forage in close association with ice-pack and during summer they mainly feed their chicks with fish (up to 95% of the preys [24]). To our knowledge no studies have described the trophic niche of snow petrels during the inter-breeding period, and there is no evidence of age and/or sex related trophic niche segregation.

Taking age into consideration is crucial when considering the consequences of Hg contamination. Indeed, several studies on vertebrates have reported breeding impairment from Hg including suppression of reproduction, reduced clutch size, poor breeding performances or unusual courtship behavior [25], [26], [27], [28], [29]. Indeed, in its methylated form, Hg is highly toxic and is known to disrupt the reproductive endocrine system [25]. For instance, in an Arctic seabird, the black-legged kittiwake *Rissa tridactyla*, a recent study has reported that Hg could interfere with the secretion of luteinizing hormone (LH) [29] a major hormone for the onset of breeding [30]. In vertebrates, the onset of breeding involves the activation of the hypothalamic-pituitary-gonadal axis (hereafter HPG axis): increasing day length activates the expression of a neuro-hormone, the Gonadotropin Releasing Hormone (GnRH) which triggers the secretion of LH from the pituitary gland. LH, in concert with follicle-stimulating hormone (FSH), promotes gonadal maturation, sex steroid secretion and in turn, sexual behaviors [30]. In black-legged kittiwakes plasma baseline LH concentrations of non-breeding males were negatively correlated to blood Hg concentrations [29]. By using the injection of exogenous GnRH, a common and powerful method to test the functionality of the pituitary through its ability to release LH [31], [32], [33], the authors suggested that Hg contamination may disrupt GnRH secretion from the hypothalamus in black-legged kittiwakes [29]. The effect of Hg on LH secretion is supported by experimental investigations: Hg-fed male rats had decreased concentrations of LH and testosterone [34]. LH secretion could thus be a potential target for Hg. In addition LH secretion is also closely related to age across vertebrates [35] including long-lived seabirds like the snow petrel [33]. Regarding our study model, the snow petrel, we therefore predict a negative relationship between blood Hg and baseline LH in the most contaminated age class. To investigate the underlying mechanism of such possible relationship, we used a GnRH challenge to test the ability of the pituitary to release LH in relation to Hg. If GnRH-induced LH concentrations are negatively correlated to blood Hg, this would indicate that Hg affects the ability of the pituitary to properly release LH.

## Materials and Methods

### Ethics statement

Animals were cared for in accordance with the guidelines of the ethics committee of the Institut Polaire Français Paul Emile Victor (IPEV) that specifically approved this study (Program no. 109, H. Weimerskirch).

### Capture, blood sampling and GnRH-challenge

The study was conducted on Adélie land (66°40′ S, 140°01′ E), Antarctica. Many snow petrels were of known age, because chicks have been banded each year since 1964 [17]. Twenty-nine males and 16 females, from 11 to 45 years old, were handled during the pre-laying period (i.e. the courtship and mating period, a few days after arrival to the colony), from 11 to 23 November 2008. Only one bird was captured at each nest and the sex of the birds was previously determined by molecular sexing [36]. Snow petrels were caught by hand and a first blood sample was collected from the alar vein with a syringe immediately after capture (mean ± S.D.: 3 min±5 s) to determine baseline plasma LH concentrations. Then, 40 birds were immediately injected with 0.1 ml of a GnRH solution in the second alar vein ([Gln8], Sigma Lot 121H04314). The GnRH was dissolved in a physiological solution to yield a dosage of 0.6 mg for 0.1 ml (about 1.5 mg kg^−1^ body mass in 1 ml of 0.9% saline solution, as validated in this species [33]). In snow petrels, a GnRH injection elicits a maximal increase of LH after 10 minutes, and this response documents the ability of the pituitary to release LH. In this species, measuring LH levels 30 minutes post-GnRH challenge can inform on an individual’s capacity to maintain elevated LH concentrations [33]. Blood samples were therefore collected from alar veins at 10 and 30 min after the GnRH injection to measure plasma LH concentrations. LH changes from 0 to 10 min post-GnRH injection [(LH_t = 10_ − LH_t = 0_)/LH_t = 0_] and LH changes from 10 to 30 min post-GnRH injection [(LH_t = 30_ − LH_t = 10_)/LH_t = 10_] were used as indicators of different individuals’ abilities to release LH. Blood samples were centrifuged and plasma (for LH assay) and red blood cells (for total Hg and stable isotopes assays) were stored at −20°C. Adults were weighted to the nearest 2 g using a spring balance and their skull length (head+bill) was measured to the nearest 0.5 mm using a caliper. In snow petrels, skull length appears to be a reliable measurement of the overall size of a bird [36] and adult scaled mass index was then calculated [37].

### Hg analysis

Total Hg was measured as described in [38] from freeze-dried and powdered red blood cells (hereafter called blood) in an Advanced Hg Analyzer spectrophotometer (Altec AMA 254). Prior and after freeze-drying, blood samples were weighed to determine the percentage of water in blood to facilitate comparisons with other studies. At least two aliquots ranging from 5 to 10 mg were analyzed for each individual and quality assessment was measured by repeated analyses of certified reference material TORT-2 (lobster hepatopancreas, NRCC; certified value 0.27±0.06 µg⋅g^−1^). Hg concentrations are expressed in µg⋅g^−1^ dry weight (dw).

### Stable isotopes analysis

The isotopic niche of snow petrels was used as a proxy of their trophic niche during the pre-breeding period, as blood cells is a metabolically active tissue that integrates periods of weeks before sampling [39], [40] and [41]. The isotopic method was validated in the southern Indian Ocean, with δ^13^C values of seabirds indicating their foraging habitats [42], [43] and their δ^15^N values increasing with trophic level [44]. Before isotopic analysis, blood samples were freeze-dried and powdered. Sub-samples of homogenized blood powder were then weighed (∼0.4 mg) with a microbalance and packed in tin containers. Relative abundances of C and N isotopes were determined with a continuous flow mass spectrometer (Thermo Scientific Delta V Advantage) coupled to an elemental analyzer (Thermo Scientific Flash EA 1112). Isotopic results are presented in the δ notation relative to Vienna PeeDee Belemnite and atmospheric N_2_ for δ^13^C and δ^15^N, respectively. Replicate measurements of internal laboratory standards (acetanilide) indicate measurement errors <0.10 ‰ for both δ^13^C and δ^15^N values. The C:N mass ratios of the samples were calculated as the ratio between the mass percentages in carbon and nitrogen. The consistently low C:N values of blood cells verified that low lipid content did not necessitate lipid extraction [45].

### Hormone assays

Plasma LH concentrations were assayed by radioimmunoassay as validated for snow petrels [33]. The lowest detectable concentration of LH was 0.05 ng⋅ml^−1^ and the intra-assay coefficient of variation was 6.2% (three duplicates). In 4 birds plasma levels were too low to measure LH.

### Statistics

All analyses were performed using R v. 2.13.1. We used GLM with normal error and identity link function to explain: (i) blood Hg concentration in relation to age, blood δ^13^C and δ^15^N, scaled mass index, and interactions with sex, (ii) blood δ^13^C and δ^15^N values in relation to age, age^2^, scaled mass index and interactions with sex (linear and/or quadratic). To address the interaction between LH, Hg and age, the dataset was split into two groups using a recently developed Bayesian approach (‘broken stick’ modelling technique [46], [47], based on beta regression analysis with ‘betareg’ package in R, [48]. This method will infer the ‘age limit’ where blood Hg concentration starts translates statistically as a change-point in the distribution of blood Hg concentration versus age. To estimate the age limit of the change-point *T_i_*, in blood Hg concentrations we used a profile likelihood approach [47]: the likelihood was computed for each age. The value of *T_i_* that maximized the likelihood was thus evaluated, and an approximate confidence interval for *T_i_* was computed with a Likelihood Ratio Test with 1 df. This method bypasses the need to manually inspect the data for identification of a change-point, which could only be uncertain in our case owing to the high variance observed in individual blood Hg concentrations. Thanks to this method we were able to distinguish a ‘break’ in Hg distribution at 23 years old in our data set. Thus we considered two groups: birds that were until 23 years old (N = 31) and birds that were more than 23 (N = 14) hereafter ‘≤23 y.o.’ and ‘>23 y.o.’, respectively), blood Hg concentrations were significantly higher in ‘≤23 y.o’ birds than in the ‘>23 y.o.’ ones (GLM, F_1,43_ = 4.8, p = 0.034). Finally (iii) absolute plasma baseline LH concentrations, LH changes from 0 to 10 minutes, and changes from 10 to 30 minutes were tested as functions of blood Hg concentration (linear) in relation to age classes (‘≤23 y.o.’ and ‘>23 y.o.’). We performed all our model selection starting from the most parameterized model that included all the variables/factors and their interactions and we removed step by step the non-significant interactions, variables or factors. Selected models were checked for assumptions and values were log-transformed when necessary, we used GLM with normal error and identity link function to explain LH concentrations in relation with Hg and interaction with sex. Values are means ± SD.

## Results

### Hg concentrations in relation to age, sex and trophic niche

**Figure 1:**
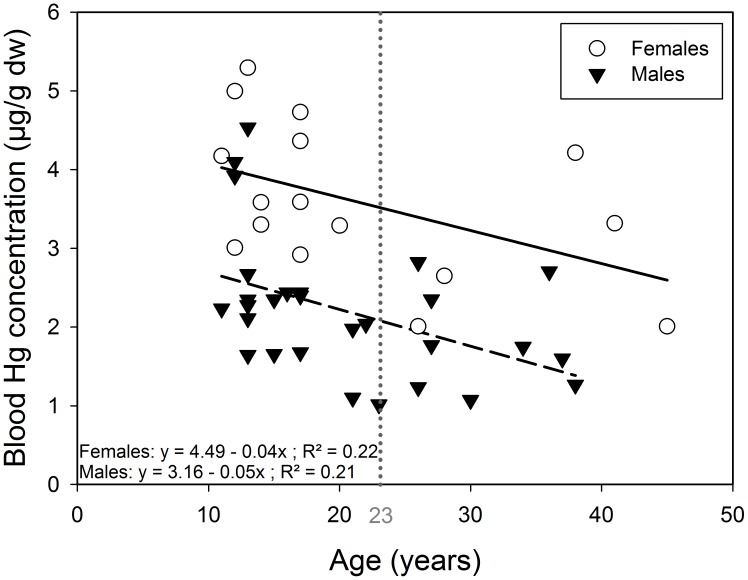
Blood Hg concentration in relation to age in pre-laying snow petrels. Hg concentration significantly decreased with increasing age in males (black triangles). For females (white circles) this relationship was close to statistical significance (P = 0.06). Solid line refers to linear regression for females and dashed line to linear regression for males. The vertical dashed line shows the change point inferred by the ‘broken sticks’ determination process for blood Hg concentration in relation with age (23 years old).

**Figure 2:**
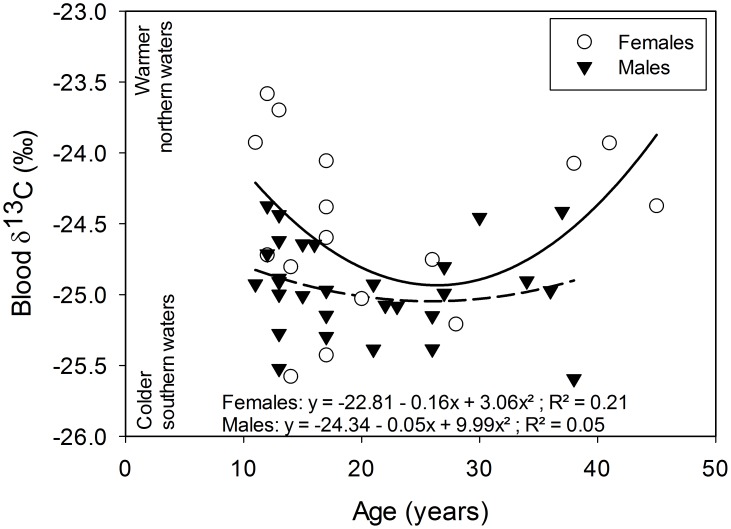
Blood δ^13^C values in relation to age in pre-laying snow petrels. δ^13^C values quadratically varied with age and this was more pronounced in females (white circles) than in males (black triangles). Solid line refers to quadratic regression for females and dashed line to quadratic regression for males.

**Figure 3:**
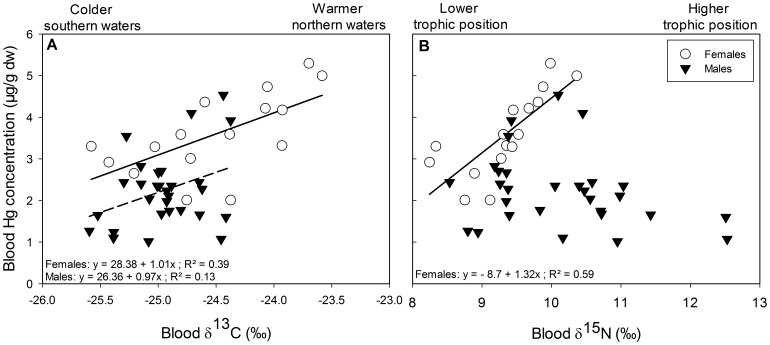
Blood Hg concentration in relation with blood δ^13^C and δ^15^N values in pre-laying snow petrels. (A) Hg concentration increased with increasing δ^13^C values for females and this relationship was close to significance for males (P = 0.06), while (B) Hg concentration increased with increasing δ^15^N values in females only (white circle). Solid line refers to linear regression for females and dashed line to linear regression for males.

In snow petrels dw blood was equivalent to 35.5±0.3% of ww blood and Hg concentration averaged 2.7±1.1 (range: 1.0; 5.3) µg⋅g^−1^ dw During the pre-laying period, blood Hg concentrations were not related to sampling date (GLM, F_1,43_ = 0.5, p = 0.488) and were higher in females than in males (**Table 1**). Blood Hg concentration decreased with increasing age in males (GLM, F_1,27_ = 7.9, p = 0.009, **Fig. 1**) and this negative relationship was only close to statistical significance in females (GLM, F_1,14_ = 4.0, p = 0.06, **Fig. 1**). Females had significantly higher δ^13^C and lower δ^15^N values than males (**Table 1**). A significant U-shaped relationship between blood δ^13^C values and age was found, i.e. young and old birds had higher δ^13^C values than middle-aged ones (**Fig. 2** **,** **Table 2**). Blood δ^15^N values were not related to age (**Table 2**). Blood Hg concentrations increased with increasing blood δ^13^C in females while in males the similar pattern was found but only close to statistical significance (**Fig. 3A** **,** **Table 3**). Regarding the relationships between blood Hg and δ^15^N values, a positive relationship was found in females only (**Fig. 3B** **,** **Table 3**). Scaled mass index was not related to blood Hg concentration, δ^13^C or δ^15^N values and interactions with sex (p>0.099 for all tests).

**Table 1:** Blood Hg concentrations, δ^13^C and δ^15^N values in pre-laying snow petrels in relation to sex.

Blood values	Males	Females	Sex differences			
			SS*	Df	F	p-value
Hg (µg⋅g^−1^ ww)	2.2±0.9 (1.0; 4.5)	3.6±1.0 (2.0; 5.3)	18.7	1,43	21.9	>0.001
δ^13^C (‰)	−25±0.3 (−25.6; −24.4)	−24.5±0.6 (−25.6; −23.6)	1.9	1,43	10.1	>0.001
δ^15^N (‰)	9.3±0.6 (8.5; 12.5)	10.1±1.0 (8.2; 10.4)	6.5	1,43	8.3	0.006

*SS = Sum of squares.

Values are means ± SD with ranges in parentheses.

**Table 2:** Modelling the effects of age (years), age^2^, sex and interactions as a function of blood δ^13^C and δ^15^N values (‰) in pre-laying snow petrels.

Dependent variable	Independent variable	SS*	Df	F	p-value
a) δ^13^C	**Age^2^**	1.0	1,32	5.0	**0.033**
	**Age**	0.9	1,32	4.5	**0.041**
	**Sex**	1.5	1,32	7.5	**>0.001**
	Age^2^×Age	0.8	1,32	4.1	0.052
	Age^2^×Sex	0.7	1,32	3.7	0.063
	Age×Sex	0.7	1,32	3.4	0.074
	Age^2^×Age×Sex	>0.1	1,32	>0.1	0.862
b) δ^15^N	Age^2^	>0.1	1,32	>0.1	0.809
	Age	>0.1	1,32	>0.1	0.876
	**Sex**	5.3	1,32	6.1	**0.019**
	Age^2^×Age	0.2	1,32	0.2	0.644
	Age^2^×Sex	0.6	1,32	0.7	0.421
	Age×Sex	0.7	1,32	0.8	0.369
	Age^2^×Age×Sex	>0.1	1,32	>0.1	0.927

*SS = Sum of squares.

**Table 3:** Modelling the relationships between blood δ^13^C, δ^15^N values (‰) and blood Hg concentration (µg⋅g^−1^ dw) in male and female pre-laying snow petrels.

Dependent variable	Sex	Independent variable	SS	Df	F	p-value
Hg	Males	δ^13^C	2.8	1,27	3.9	0.060
		δ^15^N	1.7	1,27	2.2	0.150
	Females	**δ^13^C**	5.7	1,14	9.1	**0.009**
		**δ^15^N**	8.6	1,14	20.6	**>0.001**

*SS = Sum of squares.

### Hg, age and plasma LH

**Figure 4:**
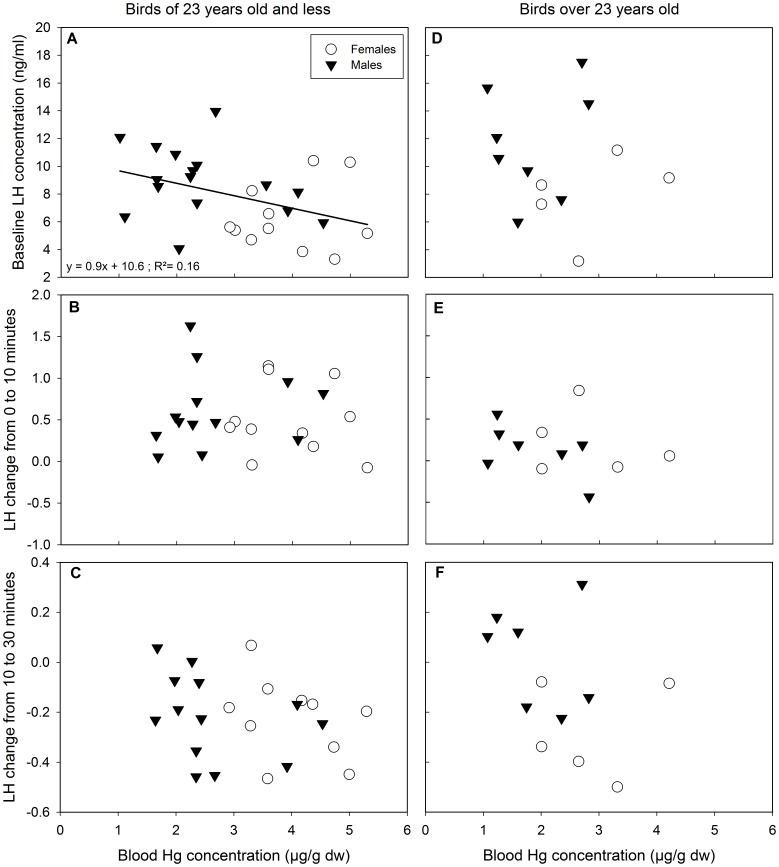
Plasma LH concentrations in relation to blood Hg concentration. In pre-laying snow petrels, baseline LH (A) decreased with increasing Hg concentration in individuals ≤23 years old (left panel) but not in individuals >23 years old (right panel, D). LH changes from 0 to 10 and from 10 to 30 minutes following GnRH challenge were not related to blood Hg concentrations in any age class (B-C-E-F). Solid line refers to linear regression for both sexes.

Following GnRH injections, absolute LH concentrations (baseline: 8.4±0.5 ng⋅ml^−1^) significantly increased (10 min: 11.5±0.8 ng⋅ml^−1^), then decreased (30 min: 9.1±0.8 ng⋅ml^−1^; generalized linear mixed model (GLMM), time as factor: F_2,61_ = 12.57, p<0.001) these LH variations were not different between sexes or age classes (≤23 y.o. versus >23 y.o.) (p>0.08 for all tests). Absolute plasma LH concentrations (baseline, after 10 and after 30 minutes) were significantly higher in males compared to females (p<0.009 for all tests) and were not related to sampling date (p>0.123 for all tests) or to scaled mass index (p>0.07 for all tests). In birds that were ≤23 y.o., plasma baseline LH significantly decreased with increasing Hg concentration in blood (**Fig. 4A**, **Table 4**) and LH changes from 0 to 10 minutes and from 10 to 30 minutes following the GnRH challenge were unrelated to blood Hg concentration (**Fig. 4B–C**, **Table 4**). In birds that were >23 y.o., plasma baseline LH concentration, LH changes from 0 to 10 minutes and from 10 to 30 minutes, were unrelated to blood Hg concentration (**Fig. 4D–E–F**, **Table 4**).

**Table 4:** Modelling the relationships between blood Hg concentration a) baseline plasma LH concentrations, b) LH change from 0 to 10 minutes and c) LH change from 10 to 30 minutes in pre-laying snow petrels ≤23 years old and >23 years old.

Independent variable: Hg	≤23 years old				>23 years old			
Dependent variable	SS	Df	F	p-value	SS	Df	F	p-value
a) Baseline LH	31	1,25	5	**0.041**	0.8	1,11	0	0.830
b) LH change from 0 to 10 minutes	0	1,21	0	0.713	0.1	1,10	1	0.429
c) LH change from 10 to 30 minutes	0	1,19	1	0.269	0.1	1,9	2	0.205

SS = Sum of squares.

## Discussion

### Hg exposure, trophic niche and age

In pre-laying snow petrels, blood Hg concentrations were in the range of concentrations found in other species from the Southern ocean [16], [49], [50] and equivalent to those reported at the same location (Adélie Land) in south polar skuas *Catharacta maccormicki* (2.2±0.2 µg⋅g^−1^ dw [51]). Blood Hg concentrations were higher in females than in males. In birds, Hg sex differences are often observed and are more likely to be related to trophic guild than other extrinsic or intrinsic factors [52]. In the present study we have an evidence of trophic guild difference between sexes; indeed stable isotopes analyses showed that foraging habitat (δ^13^C values) was positively related to blood δ^13^C values especially in females. According to the latitudinal δ^13^C gradient in oceanic waters of the Southern Ocean [42], [43], blood δ^13^C values suggest the use of a high Antarctic habitat [53]. More precisely, the δ^13^C values close to −23 ‰ depict warmer waters at more northern latitudes and the δ^13^C values close to −26 ‰ colder waters at more southern latitudes, but still within the Antarctic zone [53]. Hg distribution, and hence its bioavailability is known to be heterogeneous in the Southern Ocean [54]. The positive relationship between δ^13^C values and Hg concentrations means that Hg might be more bioavailable for snow petrels at more northern latitudes (δ^13^C values close to −23 ‰).

Trophic position, as depicted by blood δ^15^N values, was positively related to blood Hg concentration in females only. The enrichment of Hg with increasing trophic level has been described in food-webs and species communities [8], [9] but more rarely within a given species. For example, Hg enrichment with increasing δ^15^N values was found in chicks but not in adults of great skuas *Catharacta skua* [19]. In Adélie land, snow petrels feed their chicks mainly with fish [24], but the adults’ diet is still unknown. When compared to other Antarctic seabirds and their prey [53], the large range of blood δ^15^N values suggests that self-feeding breeding adults fed from a krill- to a fish-based diet, depending on individuals. The higher blood δ^15^N values of males indicate they fed more on fish than females. However and unexpectedly despite their higher trophic level, males are less Hg contaminated than females, suggesting once more that females may feed in areas where Hg is more bioavailable. This is in agreement with the δ^13^C values-Hg concentration relationship. Alternatively, detoxification processes could be more efficient in males. Noticeably, their stable isotope values indicate that the most contaminated males fed on fish at more northern latitudes than the others, suggesting again a latitudinal effect on Hg availability [54]. To summarize, females probably fed at more northern latitudes and at a lower trophic position compared to males. However, females were more Hg contaminated and Hg contamination was positively related to foraging habitat and trophic position, while in males no relationships were found. These results could be the consequence of sex-specific foraging preferences [55], [56] or maybe sex-specific physiological constraints. Indeed, females are 20% lighter than males consequently they could be more sensitive to the harsh Antarctic climate during winter and constraint to forage in more northern latitudes where Hg is more bioavailable.

Contrary to our prediction blood Hg concentration decreased with increasing age in pre-laying snow petrels, especially in males. The relationship between Hg and age in seabirds is poorly documented and often contradictory. For example, in organs involved in storage and detoxification as the liver, Hg concentration may decrease, increase, with advancing age or may not be related to age [12], [13] and [15]. In excretory tissues as feathers, Hg reaches a plateau until individuals start breeding, and then feather Hg concentration decreases [16]. Regarding blood Hg, two studies on Procellariiforms found no relationship between Hg and age [16], [50]. However in south polar skuas, breeding experience (which is tightly related to age) was negatively related to blood Hg [51], as found in our study. This pattern could originate from an age-related change in trophic niche i.e. older individuals would forage in areas where Hg is less bioavailable and/or at a lower trophic level than younger ones. Indeed, trophic niche segregations by age, has been observed in some Procellariform species [55], [56]. In our study, a quadratic relationship was found between foraging habitat (δ^13^C values) and age. This suggests that, compared to middle-aged birds, young and old individuals foraged at higher latitudes which may be more Hg contaminated. Isotopic signature of blood represents the isotopic niche during the last month preceding sampling [53]. As birds have been sampled in November (a few days after their arrival on the breeding area) those values may mirror the trophic niche used during late winter and suggest that young and old snow petrels do not use the same foraging areas than middle-aged birds at that time. And why older individuals bear lower Hg concentrations than younger birds could be the consequence of an increase in Hg excretion rate with increasing age or the selective disappearance of the most contaminated individuals over time. One may assume that Hg excretion opportunities increase with age; as older individuals had more opportunities to excrete Hg, through molting, and egg-laying for females, than younger birds. But there is no evidence for supporting such an assumption. Also, our study was cross-sectional, and since in its methylated form Hg is highly toxic, the possibility that most of the highly contaminated birds died selectively over time cannot be excluded. In Adult common loons (*Gavia immer*) blood Hg concentrations of 2–3 µg⋅g^−1^ (ww) are considered at moderate risk to effects of Hg contamination [57]. In snow petrels ww blood Hg concentrations would reach 1.6 µg⋅g^−1^ and 1.9 µg⋅g^−1^, for the most contaminated male and female respectively; these concentrations are close to the threshold level for moderate risk in common loons and snow petrels could maybe be more sensitive to lower Hg concentrations. However, in free-living birds, Hg has so far never been reported as affecting survival probabilities, even in highly contaminated areas [51], [58], [59], [60], [61].

### LH secretion in relation to age and Hg

In the present study, plasma baseline LH decreased with increasing blood Hg in individuals until 23 y.o., which were those that bore the higher concentrations of blood Hg. This negative relationship between plasma baseline LH and blood Hg could either originate from a Hg-related dysfunction of the pituitary (i.e. an inability to produce enough LH) and/or of the hypothalamus (i.e. a lack of endogenous GnRH production). Since LH changes (0–10 min and 10–30 min) following an exogenous input of GnRH were not related to Hg, this means that Hg does not disrupt the capability of the pituitary to release LH in birds until 23 y.o.: their pituitary is thus functional. This rather suggests, as found in black-legged kittiwakes, of a problem originating from the hypothalamus, which may not release enough GnRH in response to high Hg exposure [29]. However, contrary to black-legged kittiwakes [29], Hg contamination did not result in an over-release of LH post-GnRH injection in snow petrels. In black-legged kittiwakes, the over-release of LH was interpreted as an increase of GnRH receptors on the pituitary in response to the low level of GnRH secreted by the hypothalamus [29]. In snow petrels, the concentrations of blood Hg are slightly higher than in black-legged kittiwakes (2.7±1.1 µg⋅g^−1^ against 2.0±0.5 µg⋅g^−1^, respectively) and LH secretion post-GnRH injection was less pronounced in snow petrels compared to kittiwakes (averagely 1.4-fold and 2.0-fold, respectively). It is always difficult to compare different species as hazardous effects of Hg could be highly species-specific [51]: e.g. average blood Hg concentrations in brown skuas were much higher than in south polar skuas (8.2±0.2 and 2.2±0.2 µg⋅g^−1^ dw, respectively) however south polar skuas suffered from higher Hg-induced breeding failure than their closely relative [51]. Thus, it is possible that black-legged kittiwakes could be more sensitive to Hg than snow petrels and would react more strongly to slightly lower Hg concentrations. In birds that were more than 23 y.o., blood Hg concentrations were lower than in the ≤23 age class, and no relationship appeared between plasma LH and blood Hg concentrations. However sample size was low for individuals aged above 23, and such interpretation must be taken cautiously. The onset of breeding is importantly controlled by an increase of circulating LH in the plasma [62], [63], if these concentrations are not high enough individuals will be less receptive to mating behaviors [64]. In snow petrels a large proportion of young individuals do not breed each year [65], this non-breeding behavior could thus be related to Hg contamination.

### Conclusion

This study shows the importance of considering age in eco-toxicological studies. We reported an unexpected negative relationship between age and blood Hg concentrations in adult snow petrels and the use of stable isotopes suggests that this pattern could originate from an age and sex-related variation in late wintering areas, and hence Hg bioavailability. It is now possible to map migration and winter distribution of relatively small birds such as snow petrels via miniature loggers (geolocation sensors, GLS, e.g. [66]). These devices could thus open new research possibilities to confirm the influence of age on the wintering areas used by this Antarctic species and to document how migratory strategies and winter distribution can affect the uptake of contaminants. We also showed that the age of the birds and their contaminant burden could influence the secretion of a key pituitary hormone for the onset of breeding (LH). This result, together with recent findings on black-legged kittiwakes, supports the view that Hg is tightly connected to LH secretion and then can impair the decision to breed [29] and long-term reproductive success [51] in polar seabirds. Anthropogenic Hg emissions are expected to rise in the coming decades [67], probably resulting in increasing Hg exposure for Antarctic top predators. This, coupled with a wide range of environmental perturbations in Antarctic regions could exacerbate the demographic responses of top predators to Hg [51].
